# Analysis of a Pure Magnesium Membrane Degradation Process and Its Functionality When Used in a Guided Bone Regeneration Model in Beagle Dogs

**DOI:** 10.3390/ma15093106

**Published:** 2022-04-25

**Authors:** Patrick Rider, Željka Perić Kačarević, Akiva Elad, Daniel Rothamel, Gerrit Sauer, Fabien Bornert, Peter Windisch, Dávid Hangyási, Balint Molnar, Bernhard Hesse, Frank Witte

**Affiliations:** 1 Department of Prosthodontics, Geriatric Dentistry and Craniomandibular Disorders, Charité—Universitätsmedizin Berlin, Aßmannshauser Straße 4–6, 14197 Berlin, Germany; patrick.rider@botiss.com (P.R.); zpkacarevic@fdmz.hr (Ž.P.K.); 2 Botiss Biomaterials AG, Ullsteinstrasse 108, 12109 Berlin, Germany; a.elad@hotmail.com; 3 Department of Anatomy Histology, Embryology, Pathology Anatomy and Pathology Histology, Faculty of Dental Medicine and Health, University of Osijek, 31000 Osijek, Croatia; 4 CMF Surgery, Johannes BLA Hospital, 41239 Mönchengladbach, Germany; daniel.rothamel@mg.johanniter-kliniken.de (D.R.); gerritsauer@me.com (G.S.); 5 Faculté de Chirurgie Dentaire de Strasbourg, Université de Strasbourg, 8 rue Sainte-Elisabeth, 67000 Strasbourg, France; bornertfabien@gmail.com; 6 Department of Periodontology, Semmelweis University, 1769 Budapest, Hungary; peter.windisch@gmail.com (P.W.); sosefelejtemel@gmail.com (D.H.); molbal81@gmail.com (B.M.); 7 Xploraytion GmbH, Bismarkstrasse 11, 10625 Berlin, Germany; hesse@xploraytion.com

**Keywords:** NOVAMag membrane, resorbable membrane, GBR, healing, magnesium degradation, micro-CT

## Abstract

For the surgical technique of guided bone regeneration (GBR), the choice of available barrier membranes has until recently not included an option that is mechanically strong, durable, synthetic and resorbable. The most commonly used resorbable membranes are made from collagen, which are restricted in their mechanical strength. The purpose of this study is to evaluate the degradation and regeneration potential of a magnesium membrane compared to a collagen membrane. In eighteen beagle dogs, experimental bone defects were filled with bovine xenograft and covered with either a magnesium membrane or collagen membrane. The health status of the animals was regularly monitored and recorded. Following sacrifice, the hemimandibles were prepared for micro-CT (μ-CT) analysis. Complications during healing were observed in both groups, but ultimately, the regenerative outcome was similar between groups. The μ-CT parameters showed comparable results in both groups in terms of new bone formation at all four time points. In addition, the μ-CT analysis showed that the greatest degradation of the magnesium membranes occurred between 1 and 8 weeks and continued until week 16. The proportion of new bone within the defect site was similar for both treatment groups, indicating the potential for the magnesium membrane to be used as a viable alternative to collagen membranes. Overall, the new magnesium membrane is a functional and safe membrane for the treatment of defects according to the principles of GBR.

## 1. Introduction

The concept of guided bone regeneration (GBR) is based on the placement of a barrier membrane to exclude unwanted tissues and cells from a secluded bony defect. The membrane provides space for slowly proliferating bone cells to populate the defect space, which would otherwise be occupied by faster proliferating soft tissue cells [1,2]. In addition to the exclusion of unwanted tissues, the membrane creates space for undisturbed bone regeneration, protects the underlying blood clot and stabilizes the wound.

Collagen barrier membranes are currently one of the most commonly used resorbable membranes for GBR surgeries [3,4], and they demonstrate an excellent biocompatibility [5]. Due to their structure, collagen nanofibers have a good bioactive potential in bioregeneration [6].

Yet a relatively low mechanical strength means that they are susceptible to tearing or collapse into the defect void [7], which has been reported as the main drawback for using collagen membranes [8], because it does not provide sufficient volume stability at the time of bone formation [9]. In order to better control the degradation and improve the mechanical and chemical properties of collagen, new synthetic approaches have emerged that improve the properties of the collagen membrane [10,11].

There is also the potential issue of conflicting patient views, who may opt for synthetic materials.

**Figure 1:**
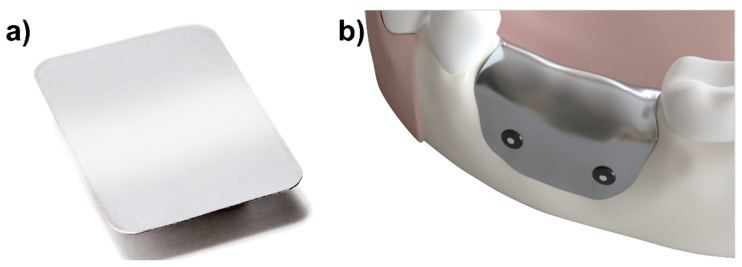
(**a**) Pure magnesium membrane (NOVAMag^®^ membrane, botiss biomaterials GmbH, Germany) used for GBR. (**b**) the magnesium membrane is positioned over the bony defect during the GBR procedure to provide a mechanical barrier between the soft and the hard tissues.

To address these issues, a new pure magnesium barrier membrane has been developed for GBR applications (Figure 1) and has previously been reported on [12]. The membrane is intended to function similarly to other degradable barrier membranes; however, due to its metallic structure, it provides better mechanical properties (than e.g., collagen) and has an initial form stability to protect the defect void from collapse.

Magnesium is a biodegradable metal that has been used for medical applications for over 100 years [13], owing to its excellent biocompatibility, bioabsorbability and biomechanical properties [14]. As it degrades, its metallic structure is converted into magnesium salts that are then resorbed by the body [15,16,17]. As a fully biodegradable metal membrane, no removal surgery is necessary, resulting in fewer surgical interventions. During its degradation under physiological conditions, hydrogen gas is released [18,19]. The release of hydrogen gas has previously been reported to form gas cavities around magnesium metal implants; however, these gas cavities are also reported to spontaneously resolve and not have a negative effect on bone regeneration [15,16,17,20].

As with other degradable membranes, it is important that the resorption rate enables a sufficient barrier between the soft and the hard tissues during the initial healing phase, but also for it to be fully removed from the site once it is no longer needed. The resorption time should not exceed 6–12 months; otherwise, the benefits provided by using a resorbable material might be lost [21].

Studies performed using collagen membranes give an indication for the optimal functional lifespan of a barrier membrane. An in vivo study using rat calvarial defects performed by Kim et al. reported that the collagen membrane, Bio-Gide^®^, remained intact after 2 weeks, but after 4 weeks, it had lost its barrier function [22]. A study by von Arx et al. performed in rabbit tibias showed that the collagen membrane was intact after 2 weeks but reduced in size after 6 weeks healing. After 12 weeks, no differentiation to the host collagen could be made [23].

In a GBR canine model, collagen membrane degradation has been reported with more varied rates, even when using the same type of collagen membrane. Ivanovic et al. used a double layer technique to prolong functionality and reported the presence of residual membrane material at 12 weeks post-implantation [24]. Rothamel et al. reported that the membrane was resorbed between 4 and 8 weeks post-implantation [25], whilst Zubery et al. reported complete degradation of the membrane at different stages of the study, ranging from the first (8 weeks) to the last (24 weeks) time point [26].

Based on these studies reporting the degradation of a collagen membrane, it can be assumed that the barrier membrane must function for a minimum period of 2–4 weeks. After this point, some of these studies have reported differing degrees of degradation and loss of barrier function; however, all resulted with a successful regenerative outcome.

The magnesium membrane and all the ideal qualities for a barrier membrane were previously reported on [12]. In this article, the application of a pure magnesium barrier membrane to treat GBR defects in a canine model is reported on. The success of the membrane is determined by comparison to a standard collagen membrane. Magnesium membrane degradation and regenerative outcome are assessed using µCT.

## 2. Results

### 2.1. Post-Surgical Follow-Up

As expected, for the 2 week period post-surgery (preparatory and implantation), the dogs showed signs of acute inflammation and pain. For the duration of the study, weight variations of the animals occurred as expected and remained within the anticipated ranges for beagle dogs under the conditions of this kind of study. All animals survived until their scheduled sacrifice. Following the teeth extraction surgery (preparatory phase), some dogs required additional antimicrobial treatment or bone sequestrum removal; however, all sites ended up with a good healing result.

Post-implantation (experimental phase), eight animals required an additional intervention, such as additional chlorhexidine rinsing or re-suturing; however, all sites ended up healing well. Additional interventions were mainly caused by swelling associated with magnesium membrane treated sites; however, in two of the eight animals, additional intervention was also required for collagen membrane treated sites. Most observations of swelling or lesions present at the magnesium membrane treated sites were reported during the scheduled surgical wound re-evaluation at 28 ± 2 days. Around this timepoint, 13 magnesium membrane treated sites in a total of 7 dogs reported observations of swelling. Two of these sites also reported the presence of lesions. After an additional treatment of chlorhexidine rinsing (for 3 dogs) and a varying healing period between 3 and 10 days, there were no abnormal findings reported by the veterinarian.

At the next scheduled re-evaluation (42 ± 2 days), one additional magnesium membrane-treated site was reported to have swelling and a lesion, both of which resolved after 10 days. Another surgical site in another dog was also observed to be open, however without the presence of swelling, and it resolved itself after 10 days. The redness of the surgical site was reported for four dogs and did not occur in conjunction with any swelling. Of these dogs, two were observed to have one site treated with magnesium membrane that had a slight redness. The slight redness was reported at only one timepoint for each dog (at day 36 and 43, respectively). Another dog was reported to have a slight redness for both magnesium membrane-treated sites. This was reported over a prolonged period of time, which was mentioned at day 36 and 52 for both sites; however, no abnormal findings were reported at day 57. The last animal with reported redness was observed to have a small red spot (3 mm in diameter) at a collagen membrane-treated site. The small red spot was reported after the healing of a lesion at the same site.

### 2.2. Micro-CT

**Figure 2:**
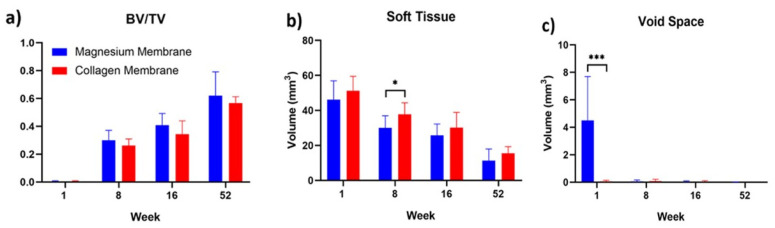
Volumetric measurements of GBR defects treated with either a magnesium membrane (blue) or collagen membrane (red): (**a**) New Bone Volume/Total Defect Volume; (**b**) Soft Tissue Volume; (**c**) Void Space Volume. Standard deviation and statistical significance are shown. *p* ≤ 0.05 is represented by “*” and *p* ≤ 0.001 is represented by “***”.

The results of the measured µCT parameters are shown in Table 1 and visually compared in Figure 2. One week post-implantation, the measured µCT parameters of new bone and soft tissue appear similar between the magnesium and collagen membrane groups. At this timepoint, the only significantly different value was the volume of the void space (*p* < 0.001). This developed in the magnesium membrane group due to the release of hydrogen gas during the magnesium corrosive process.

At 8 weeks post-implantation, the void space for the magnesium membrane had been resorbed and was no longer significantly different to that measured for the collagen membrane. There was no significant difference between the new bone volume between each group; however, there was more soft tissue present in the collagen membrane group (*p* ≤ 0.05) than in the defects treated with the magnesium membrane. The ratio of bone volume to total defect volume was comparable between the two groups (0.30 ± 0.07 for the magnesium membrane-implanted defects compared to 0.26 ± 0.05 for the collagen membrane-implanted defects).

By 16 weeks, there were no significant differences between either of the groups for any of the measured parameters. The average soft tissue volume remained slightly higher in the collagen membrane treated defect sites (30.15 ± 8.75 mm^3^ for the collagen group compared to 25.74 ± 6.49 mm^3^ for the magnesium group), although it was non-significant. The bone volume to total volume ratio appears to be slightly higher for the magnesium membrane-treated defects (0.41 ± 0.09 mm^3^) compared to (0.34 ± 0.10 mm^3^) for the collagen group, but again, it was non-significant.

For the 52-week timepoint, the new bone volume and soft tissue volume appear to be slightly lower for the magnesium membrane-treated defects, although this is related to the measured total defect volume being slightly lower than that of the collagen membrane-treated defects. Similarly, for the 16 week timepoint, the bone volume to total volume ratio appears to be slightly higher for the magnesium membrane defects, 0.62 ± 0.17 mm^3^ compared to 0.57 ± 0.05 mm^3^ for the collagen membrane-treated group.

**Figure 3:**
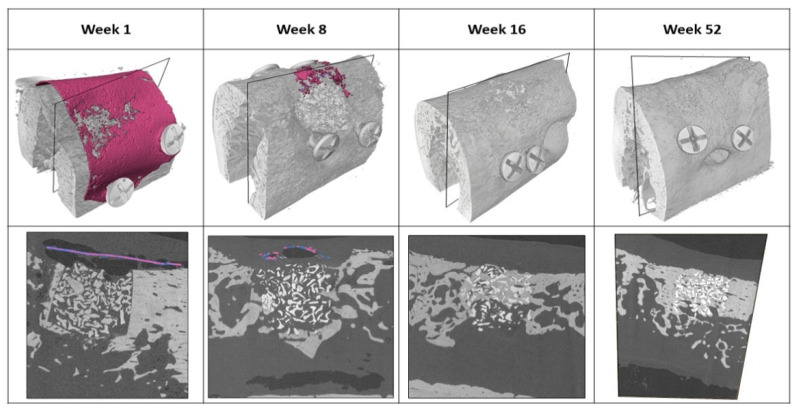
Reconstructed µCT images showing the residual metallic magnesium membrane (indicated in pink and blue magnesium salts). Only a minor amount of the metallic magnesium is left after 8 weeks and is completely corroded at 16 weeks (in 11/12 samples). At 52 weeks after implantation, no residual of the remaining magnesium membrane could be observed, whilst the surrounding bone of the defect has fully integrated bone substitute material.

The implanted magnesium membranes were successfully detected and segmented. The magnesium metal was denser than the surrounding tissue, thus enabling it to be identified. Remnants of the metallic magnesium membrane could be identified and separated from other objects in the 3D gray-scale images. Representative images of the segmented magnesium membranes and the surrounding bone tissue at each timepoint are shown in Figure 3. In only one of the samples from the 16-week timepoint (n = 12) could remnant magnesium metal and salty phase still be detected. At the 52-week timepoint, there was no metallic magnesium present at any of the defect sites (n = 4). At the one week timepoint, metallic magnesium, magnesium salts and small gas cavities are shown to seclude the overlying soft tissue from the bony defect.

**Figure 4:**
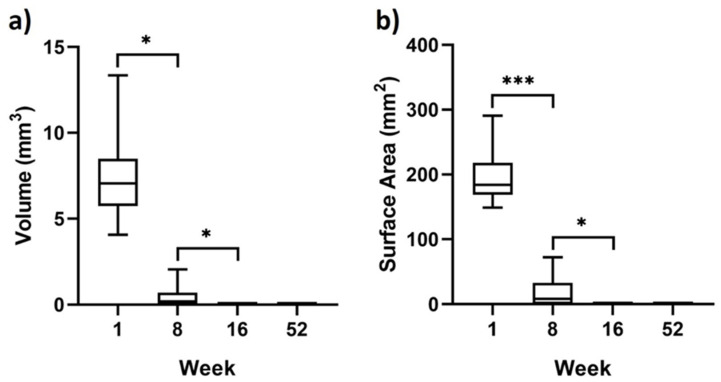
Box and whisker diagrams of (**a**) volume and (**b**) surface area measurements of the magnesium membrane remnants after implantation. Range and mean values are shown. Statistical significance is shown as *p* ≤ 0.05 represented by “*” and *p* ≤ 0.001 represented by “***”.

Measurements of the residual magnesium are shown in Figure 4. These measurements indicated an average total volume of residual magnesium metal at 1 week to be 7 ± 2 mm^3^. This average decreases significantly by week 8 to 0 ± 1 mm^3^ (*p* < 0.001) and then further to 0 ± 0 mm^3^ at week 16 (*p* < 0.05). Surface area measurements of the magnesium metal measured an average area of 198 ± 38 mm^2^ at week 1. This significantly dropped to 17 ± 22 mm^2^ at week 8 (*p* < 0.05) and then further to 0 ± 0 mm^2^ by week 16 (*p* < 0.05). All of the magnesium membranes appeared to have completely degraded at the 52-week timepoint, with both surface area and total volume measured at 0 ± 0 mm^2^ and 0 ± 0 mm^3^, respectively. Although not indicated by the surface area and volume measurements, small remnants of the magnesium membrane were still visible in one of the samples at 16 weeks.

Gas pockets formed as the magnesium metal degraded, which are visible around the membrane at the 1 week timepoint (Figure 3). The gas pockets were predominantly situated between the overlying soft tissue and the magnesium membrane upper surface, although some gas pockets were visible under the membrane as well. After 8 weeks, the majority of the gas pockets have been resorbed by the tissue, and at 16 weeks post-implantation, there are no remaining gas pockets in the vicinity of the defect site.

## 3. Discussion

A magnesium membrane has been investigated for its use in GBR surgeries. Applied to GBR defects in beagle dogs, the membrane has shown a comparable efficiency to that of a standard collagen membrane.

The first important outcome of this study was that the animals had a satisfactory general condition for the duration of the study when a magnesium membrane was applied in a GBR setting. Post-implantation monitoring showed a limited number of healing irregularities, such as swelling and lesions that were more frequently occurring in the magnesium membrane group. Although additional intervention was required for some of these animals, in all cases, the conditions stabilized or healed well without affecting the regenerative outcome. Both the magnesium membrane and the collagen membrane treatment groups did not present signs of a chronic inflammation reaction such as prolonged redness, swelling, pain and loss of function. Over the course of the study, the dogs maintained a healthy weight, which demonstrates a lack of pain and the preservation of function.

Signs of acute inflammation such as redness and swelling during healing are an expected potential outcome of GBR surgery. This was observed at both magnesium membrane-treated sites and collagen membrane-treated sites. In the sites treated with magnesium membrane, this phenomenon can be explained by the perfusion of magnesium ions into the soft tissue after the degradation of the magnesium membrane [27]. In a retrospective study of the clinical outcomes and complications of biodegradable magnesium screws in humans, similar observations were made for soft tissue complications [28]. However, this was also shown to be a short-term tissue reaction, as was the case in the current study. Overall, soft tissue complications were expected in both the magnesium membrane group and the collagen membrane group, but they did not affect the success of regeneration.

In this study, degradation of the magnesium membrane and its influence on the regenerative outcome have been evaluated using µCT. The µCT data show that the magnesium membrane was initially stable and remained largely intact 1 week post-implantation. Between 1 week and 8 weeks, the membrane underwent significant degradation that continued until week 16, when all but one of the membranes had completely degraded (Figure 3 and Figure 4).

As magnesium metal degrades, it produces hydrogen gas [19], the presence of which has been linked to a moderate inflammation reaction [28,29]. During the period between 1 and 8 weeks, the magnesium membrane experienced its largest change in volume; hence, it produced the largest volume of hydrogen, which correlates with instances of swelling that were primarily reported by the veterinarian at a 28 ± 2 days (4 weeks) post-implantation evaluation. After a maximum period of 10 days, the reported swellings had resolved, which could indicate a reduction in hydrogen gas production.

Hydrogen gas released by the degrading magnesium can lead to the formation of gas cavities around magnesium implants. These were visible around the magnesium membrane at the 1 week and 8 week timepoints (Figure 3). Nevertheless, previous studies have also reported that gas cavity formation has been followed by their spontaneous regression, and that new bone formation had not been negatively affected [15,16,17,20]. This is supported by the current study, as at the first timepoint (1 week), there was significantly more void space measured within the magnesium membrane group compared to the collagen membrane group. As the magnesium continued to degrade, the void space disappeared and remained non-significantly different to that of the collagen membrane in the subsequent timepoints (8, 16 and 52 weeks). Despite the formation of gas cavities around the magnesium membrane during the early timepoints of the study, the relative volume of new bone within the defect space consistently remained non-significantly different between defects treated with either the magnesium membrane or the collagen membrane.

The ideal degradation rate for a resorbable GBR membrane should support the regeneration of the periodontium by secluding the defect site from unwanted tissues, but it could also fully and rapidly remove the membrane once its function is no longer required. To establish an ideal degradation rate, it is possible to compare the magnesium membrane to collagen membranes, which are a popular choice for GBR surgeries [3].

There are very few studies available that directly include the degradation of collagen membranes in vivo as a specific outcome; however, three studies were found where the degradation and integration of a collagen membrane were evaluated for a similar canine defect model that was used in this study [24,25,26]. In the current study, the degradation of the magnesium membrane was monitored using µCT, whilst for the other studies, collagen membrane degradation was evaluated histologically. Although different timepoints were chosen, these studies give an indication of a comparable degradation rate.

Using a study by Rothamel et al. as a reference for collagen membrane degradation, both the magnesium and collagen membranes had an early onset of degradation that was noted at the first post-implantation timepoint; 1 week in this study for the magnesium membrane and 4 weeks for the collagen [25]. After 8 weeks, both membranes had undergone extensive degradation, with few remnants remaining. At the next sequential time point, which was 16 weeks in this study and 12 weeks for the collagen membrane, neither membrane had any visible remnants remaining. This would indicate that the degradation rates of both membranes were similar.

µCT analysis of the magnesium membrane indicated that at one week post-implantation, the metallic structure had begun to develop corrosion pits and holes, although the majority of the membrane remained intact. By week 8, even though the metallic structure of magnesium had almost completely corroded away, the bone grafting material remained in place. This is shown, as no bone substitute material can be observed outside the initially drilled bone defect. A potential reason for this is the formation of magnesium salts and hydrogen gas development during the magnesium metal degradation process, which could maintain a seclusion of the defect site [19].

This phenomenon has previously been reported on for the magnesium membrane, where its degradation kinetics were studied in a minipig model [12]. It was shown that as the membrane degraded, the resultant magnesium salt layers and gas cavities provided a secondary phase to the barrier functionality of the barrier membrane. This affect can be clearly seen at the 1 week timepoint of the segmented µCT scans (Figure 3).

## 4. Materials and methods

### 4.1. Test Item

The Test Article to be evaluated in this study is a magnesium membrane (NOVAMag^®^ membrane, botiss biomaterials GmbH, Berlin, Germany) that is produced at biotrics bioimplants AG (Berlin, Germany) from pure magnesium (99.95%). The final dimensions of the membrane are 30 × 40 mm with rounded corners that have a 4 mm radius, a thickness of 140 µm, and a weight between 245 and 330 mg. The membrane can be cut using a pair of scissors before being bent to shape and placed over the defect. It is required that the membrane be fixed into position from both the buccal and oral sides.

### 4.2. Animals and Anesthesia

In total, 20 adult male beagle dogs (*Canis familiaris*) were used in this study, which was performed at the Charles River Laboratories, Montreal, ULC. The investigatory study was approved by the Testing Facility’s Institutional Animal Care and Use Committee (IACUC). The testing facility is also accredited by the Association for the Assessment and Accreditation of Laboratory Animal Care (AAALAC) and the Canadian Council on Animal Care (CCAC). Cohorts of six animals were assigned to 1 week, 8 weeks and 16 weeks timepoints. The remaining two animals were available as spares should there be any morbidity or mortality associated with the investigation. As these spare animals were not needed, they were transferred to a 52-weeks cohort.

Two surgeries were performed as part of this study: a preparatory tooth extraction surgery and the experimental implantation surgery. Prior to both surgeries, the animals underwent general anesthesia using an injection composed of a mix of Buprenorphine, Acepromazine and Glycopyrrolate administered intramuscularly. Anesthesia induction for tracheal intubation was achieved with Propofol injected intravenously via a catheter in a vessel of the left or right cephalic or saphenous vein. Upon induction of anesthesia, the subject animal was intubated and supported with mechanical ventilation. Isoflurane in oxygen was administered to maintain a surgical plane of anesthesia, and Propofol was injected intravenously as needed to improve the efficacy of the anesthesia.

To achieve local anesthesia for teeth extraction and implantation procedures, as well as manage pain after surgery, 0.8–1.2 mL of Lidocaine mixed with Epinephrine 1:50.000 was administered in each side of the lower jaw. For teeth extraction surgeries, local anesthesia was also administered in each side of the upper jaw.

### 4.3. Surgery

The procedure was performed in two phases: a preparatory and an experimental phase. The preparatory phase involved the surgical extraction of four teeth on each side of the jaw, from the mandibular second premolar to the first molar. The corresponding teeth on the upper jaw were also extracted. Teeth extraction was followed by wound closure and suturing of the upper jaw, whilst the lower jaw remained open during a healing period of 12 ± 2 weeks. Daily oral cavity flushing was performed for 13–14 days post extraction. Sutures were removed from the upper jaw after 2 ± 1 weeks.

For the experimental phase surgery, two independent bone defects were created on each side of the lower jaw. The defects were filled with a bone substitute material (Bio-Oss^®^, Geistlich, Wolhusen, Switzerland) and covered with either a magnesium membrane or a control collagen membrane (Bio-Gide^®^, Geistlich).

**Figure 5:**
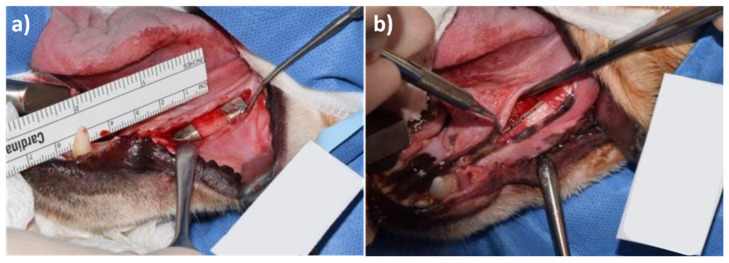
Surgical placement of (**a**) magnesium membrane and (**b**) collagen membrane in a GBR model in the lower left jaw of beagle dogs. In both images, two treatment sites are visible.

Membranes were not allocated randomly; however, an even distribution across the different sides of the lower mandible was a control of bias. Each membrane was fixed with 4 titanium screws (1.5 mm × 3 mm ProFix titanium screws, Osteogenics); 2 on the buccal side and 2 on the lingual side, followed by wound closure with sutures. Representative photos of the magnesium membrane and collagen membrane after implantation are displayed in Figure 5.

Daily oral cavity flushing was performed for 6 days post-implantation for the 1 week cohort and for 14 days post-implantation for the 8, 16 and 52-week cohorts. Sutures were removed 2 ± 1 weeks post-implantation. Upon euthanasia, hemimandibles were extracted and stored individually in 100% ethanol and kept refrigerated between 4 and 8 °C.

### 4.4. Veterinary Intervention and Care

For the duration of the study, the animals were monitored and observed (cage side observation) at least twice a day by a trained professional. The animals’ health status was followed up by a veterinarian team, as necessary.

Post-operative examinations carried out by the veterinarian team were performed under anesthesia using Propofol. Scheduled examinations post-implantation occurred three times in the first week (day 1, 3, and 7), once a week for the following three weeks (approximately day 14, 21, and 28), and thereafter once every 2 weeks (approximately day 42, 56, 70, 84, and 90), or until the day of scheduled sacrifice. The animals were weighed prior to the teeth extraction surgery, the implantation surgery and sacrifice, as wells as during veterinarian follow-ups.

### 4.5. Micro-CT Collection and Reconstruction

Prior to histological processing, each explanted hemimandible was scanned using a Nikon XTH 225 ST Micro CT scanner (Nikon, Chiyoda, Tokyo, Japan). Images were then used to reconstruct a 3D image of each implanted site. The reconstructed µCT data had a 16-bit volume and a 10 μm isotropic voxel size. Each scan contained 4 titanium screws that held the membrane in place. Where possible, each scan was used to calculate: the new bone volume/ total defect volume (BV/TV), soft tissue volume, void volume, and residual magnesium metal.

**Figure 6:**
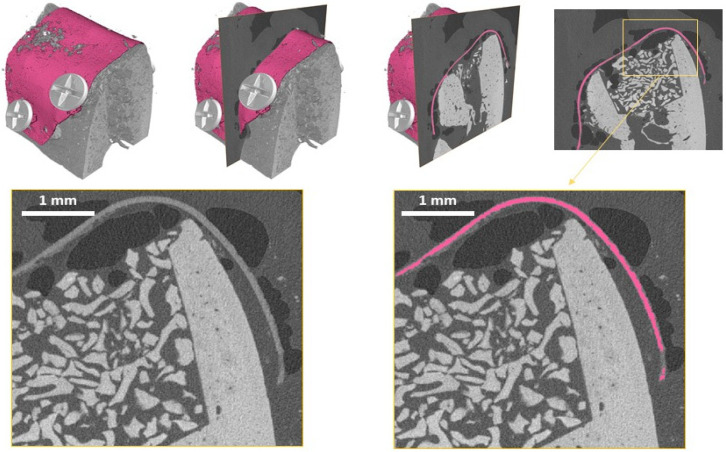
Representative images of the segmented magnesium metal membrane (pink) and virtual slices (gray) at the 1-week timepoint to illustrate the allocation of remnant metallic magnesium to gray values and image properties. Small gas cavities can be seen around the membrane, which are resultant from hydrogen gas development during the degradation process. Magnesium salts can also be seen retaining the shape and position of the membrane and are distinguished from the metallic magnesium using the employed segmentation technique within the AVIZO software (Thermo Fisher Scientific, USA).

Further analysis was performed to determine the surface area and volume of the magnesium metal. To quantify the morphology of the magnesium membrane, the membrane had to be segmented within the CT scan volume. The data were loaded into AVIZO software (Thermo Fisher Scientific, Waltham, MA, USA), and metallic remnants of the magnesium membrane were segmented using the Segmentation toolbox of AVIZO. Segmentation was achieved by combining manual segmentation steps together with region-growing approaches. The results of the obtained mask for the metallic magnesium membrane and its comparison to the original gray-scale images are demonstrated in Figure 6. This approach was used to differentiate between the remaining magnesium metal and the magnesium salts which retain the shape and position of the magnesium membrane.

The segmented volumes were loaded into MatLab (MATLAB and Statistics Toolbox Release 2018b, The MathWorks, Inc., Natick, MA, USA), and every dataset was analyzed toward its volume and surface, its surface to volume ratio as well as the number of magnesium membrane fragments. Furthermore, the number of magnesium fragments within each scan was analyzed in terms of fragment size by performing a connected component analysis.

### 4.6. Statistical Analysis

Statistical analysis was performed by grouping the implants according to their material and implant duration. The regeneration of the defect was analyzed using unpaired t-tests to identify statistically significant differences for the parameters BV/BT, soft tissue volume, and void space volume between the tested groups at each timepoint. Standard deviation and statistical significance are shown. *p* ≤ 0.05 is represented by “*” and *p* ≤ 0.001 is represented by “***”. To evaluate the degradation of the magnesium membrane, unpaired *t*-tests were used to identify statistically significant differences between the magnesium remnant surface area and volume between each sequential time point. Statistical analysis was performed using GraphPad Prism 8.1.2 Software.

## 5. Conclusions

A pure magnesium barrier membrane has been investigated as an alternative barrier membrane to be used in GBR treatment. Applied to GBR defects created in beagle dogs, veterinarian reporting and µCT analysis showed that the magnesium membrane produced a normal healing response, had a good regenerative outcome, and degraded at a rate similar to that of a collagen membrane. Although more swelling was reported for magnesium membrane-treated sites, this did not affect the regenerative outcome, and overall, there were no indications of a chronic inflammation reaction. Bone volume within the defect site remained similar to that of defects treated with a collagen membrane throughout the duration of this study. In conclusion, the results of this study indicate that the pure magnesium membrane is an effective barrier membrane suitable for GBR treatments.

## Figures and Tables

**Table 1:** µCT volume measurements for GBR defects treated with either a magnesium membrane or collagen membrane.

Week	Membrane	No. of Treated Defects	Volume				
			Total Defect (TV)	New Bone (BV)	Soft Tissue	Void	BV/TV
			(mm^3^)	(mm^3^)	(mm^3^)	(mm^3^)	
1	Magnesium	12	76.92 ± 9.41	0.34 ± 0.30	46.21 ± 10.47	4.51 ± 3.19	0.00 ± 0.00
	Collagen	12	80.15 ± 11.16	0.40 ± 0.30	51.20 ± 8.31	0.07 ± 0.07	0.00 ± 0.00
8	Magnesium	12	59.93 ± 10.89	17.71 ± 4.34	30.02 ± 6.92	0.06 ± 0.10	0.30 ± 0.07
	Collagen	12	74.73 ±9.73	19.65 ± 4.72	37.72 ± 6.62	0.08 ± 0.13	0.26 ± 0.05
16	Magnesium	12	64.14 ± 8.85	25.93 ± 5.02	25.74 ± 6.49	0.05 ± 0.05	0.41 ± 0.09
	Collagen	12	65.97 ± 7.57	22.63 ± 6.72	30.15 ± 8.75	0.06 ± 0.06	0.34 ± 0.10
52	Magnesium	4	47.89 ± 5.94	29.17 ± 5.81	11.32 ± 6.63	0.01 ± 0.01	0.62 ± 0.17
	Collagen	4	62.31 ± 2.35	35.37 ± 2.88	15.42 ± 3.90	0.00 ± 0.00	0.57 ± 0.05

## Data Availability

The data presented in this article are available on request from the corresponding author.
